# Cell proliferation effect of deep-penetrating microcavity tandem NIR OLEDs with therapeutic trend analysis

**DOI:** 10.1038/s41598-022-15197-4

**Published:** 2022-06-29

**Authors:** Yongjin Park, Hye-Ryung Choi, Yongmin Jeon, Hyuncheol Kim, Jung Won Shin, Chang-Hun Huh, Kyoung-Chan Park, Kyung-Cheol Choi

**Affiliations:** 1 School of Electrical Engineering, Korea Advanced Institute of Science and Technology (KAIST), 291 Daehak-ro, Yuseong-gu, Daejeon, 34141 Republic of Korea; 2 Department of Dermatology, Seoul National University College of Medicine, Seoul National University Bundang Hospital (SNUBH), Seongnam, 13620 Republic of Korea; 3 Department of Biomedical Engineering, Gachon University, 1342 Seongnam-daero, Sujeong-gu, Seongnam-si, 13120 Gyeonggi-do Republic of Korea

**Keywords:** Cell biology, Optics and photonics

## Abstract

Long wavelengths that can deeply penetrate into human skin are required to maximize therapeutic effects. Hence, various studies on near-infrared organic light-emitting diodes (NIR OLEDs) have been conducted, and they have been applied in numerous fields. This paper presents a microcavity tandem NIR OLED with narrow full-width half-maximum (FWHM) (34 nm), high radiant emittance (> 5 mW/cm^2^) and external quantum efficiency (EQE) (19.17%). Only a few papers have reported on biomedical applications using the entire wavelength range of the visible and NIR regions. In particular, no biomedical application studies have been reported in the full wavelength region using OLEDs. Therefore, it is worth researching the therapeutic effects of using OLED, a next-generation light source, and analyzing trends for cell proliferation effects. Cell proliferation effects were observed in certain wavelength regions when B, G, R, and NIR OLEDs were used to irradiate human fibroblasts. The results of an in-vitro experiment indicated that the overall tendency of wavelengths is similar to that of the cytochrome c oxidase absorption spectrum of human fibroblasts. This is the first paper to report trends in the cell proliferation effects in all wavelength regions using OLEDs.

## Introduction

As the public’s interest in health in the twenty-first century has increased dramatically, a variety of wearable devices such as watches^1,2^, sensors^3–5^, and healthcare systems^6–8^ have been developed as future-type electronic devices. Thus, the wearable market is growing rapidly and becoming deeply embedded in human life. In particular, the market for wearable devices and photomedicine, which can generate good synergistic effects, has been growing rapidly in recent years. The photomedicine area is classified as photobiomodulation (PBM) and includes various healing applications, such as jaundice healing, wound healing, and the treatment of acne through relatively weak energy doses (< about 10 J/cm^2^). PBM is the application of red or near-infrared light to stimulate, repair, regenerate, and protect tissue. It also includes photodynamic therapy (PDT), which destroys cells using rather strong energy doses (> about 10 J/cm^2^) for pain relief^9^, skin cancer treatment, and bacteria eradication^10^. In phototherapy, the skin penetration depth of any light depends on the absorption and scattering of the skin cells. In general, long wavelengths can penetrate deeply into the skin^11^, and such wavelengths are applied in numerous fields. Therefore, research has been conducted using lasers or light-emitting diodes(LEDs) with wavelengths above 600 nm for practical application^12–17^. However, because these lasers or LEDs are pointy and bulky light sources, there are some disadvantages, such as nonuniform irradiation, heat generation, and space–time constraints. For this reason, research using micro-LEDs^18,19^ and OLEDs^20–24^ has been actively reported. In this study, therapeutic effect was confirmed when OLEDs were used in the form of surface light sources that compensate for the shortcomings of existing light sources. In this paper, a NIR wavelength that can penetrate deeper was used because the scattering coefficient by a skin cell is lower than that of the red regions. Prior to that, it is necessary to consider the domain range of the NIR wavelength. There are many different viewpoints about where the NIR range begins, and it is difficult to distinguish accurately^25^. However, considering the luminosity curve of humans, the intensity is nearly zero over 700 nm^26^. It may be perceived as NIR as a bio-medical region and not the display. As in the bio-medical field, a wavelength in a region of over 700 nm can be considered as NIR wavelengths.

Because of the superiority of NIR wavelengths, various methods of research have been reported for the development of NIR OLEDs. However, for solution-based NIR OLEDs, their broad FWHM, insufficient radiant emittance for application to light therapy, and unstable operational lifetime are major disadvantages^27,28^. Also, NIR OLEDs fabricated by synthesizing the emitting layer have the disadvantages that the synthesis process is complicated and their lifetime is not guaranteed^29–32^. This paper proposes a vacuum-deposited NIR OLED by combining micro-cavity and tandem structures. NIR OLEDs have a narrow FWHM (34 nm), sufficient radiant emittance (> 5 mW/cm^2^) for application to phototherapy, and a stable operational lifetime. Moreover, they are simple to manufacture with an emission peak of 710 nm to 770 nm. NIR OLEDs with various emission wavelengths and visible (blue, green, red) OLEDs were tested on human fibroblasts to confirm the cell proliferation effects at each wavelength.

## Results

### Optimization process of tandem structure

NIR OLEDs were simply fabricated using a red emitter, Ir(piq)_3_ which has a broad photoluminescence (PL) peak from 550 to 800 nm, as shown in Fig. S1. NIR OLED can be implemented simply through a microcavity structure with reflective anode and cathode. However, due to low PL intensity in the NIR region of the red emitter, a tandem structure was applied to fabricate an NIR OLED with complementary advantages. The tandem structure boosts the intensity and lifetime by stacking multiple emitting layers connected to a charge generation layer (CGL)^33^. The CGL is the most important layer of the tandem structure in that it provides electrons and holes to adjacent emitting layers. In general, there are several types of CGLs, including p–n junctions, which are composed of n-doped ETL/p-doped HTL, and an n-doped ETL/metal oxide junction^33–44^. As n-type dopants, metal or metal-based compounds, such as Li, LiH^37^, LiNH_2_^38,39^, Mg^41^, and Cs_2_CO_3_^40,42,44^, are used. In this study, a Li-based n-doped ETL/metal oxide (MoO_3_) structure was used for the CGL to optimize the tandem structure. A single reference OLED and a tandem OLED were manufactured to compare their electrical properties. The structure of the single OLED had the following configuration: indium tin oxide (ITO)/molybdenum(VI) oxide (MoO_3_, 10 nm)/N,N′-Di(1-naphthyl)-N,N′-diphenyl-(1,1′-biphenyl)-4,4′-diamine (NPB, 40 nm)/Bebq_2_: Ir(piq)_3_ (30 nm, 8wt %)/Tris(8-hydroxyquinoline)aluminum (Alq_3_, 20 nm)/Liq (1 nm)/Al (100 nm). The tandem OLED multi-layer structure had the following configuration: ITO/MoO_3_ (10 nm)/NPB (40 nm)/Bebq_2_: Ir(piq)_3_ (30 nm, 8wt %)/Alq_3_: LiH (20 nm, × wt%)/MoO_3_ (10 nm)/NPB (40 nm)/Bebq_2_: Ir(piq)_3_ (30 nm, 8wt %)/Alq_3_: LiH (20 nm, × wt%)/Liq (1 nm)/Al (100 nm); device 1: (x = 1); device 2 : (x = 2); device 3: (x = 5); device 4: (x = 10); device 5: (x = 50). Figure S2 shows the schematic structure of the single and tandem devices.

Before confirming the electrical and optical performances, devices were manufactured using Liq as an n-type dopant. When Liq was doped into Alq_3_ at various concentrations, it was unable to serve as an n-type dopant. Because the structure formed with a quinoline-based organic material is complex, it did not decompose during thermal evaporation. It was difficult to supply charges from the CGL to each emitting layer due to the energy barrier with the adjacent layer. Hence, devices did not show any improvement over the single reference device shown in Fig. S3.

**Figure 1:**
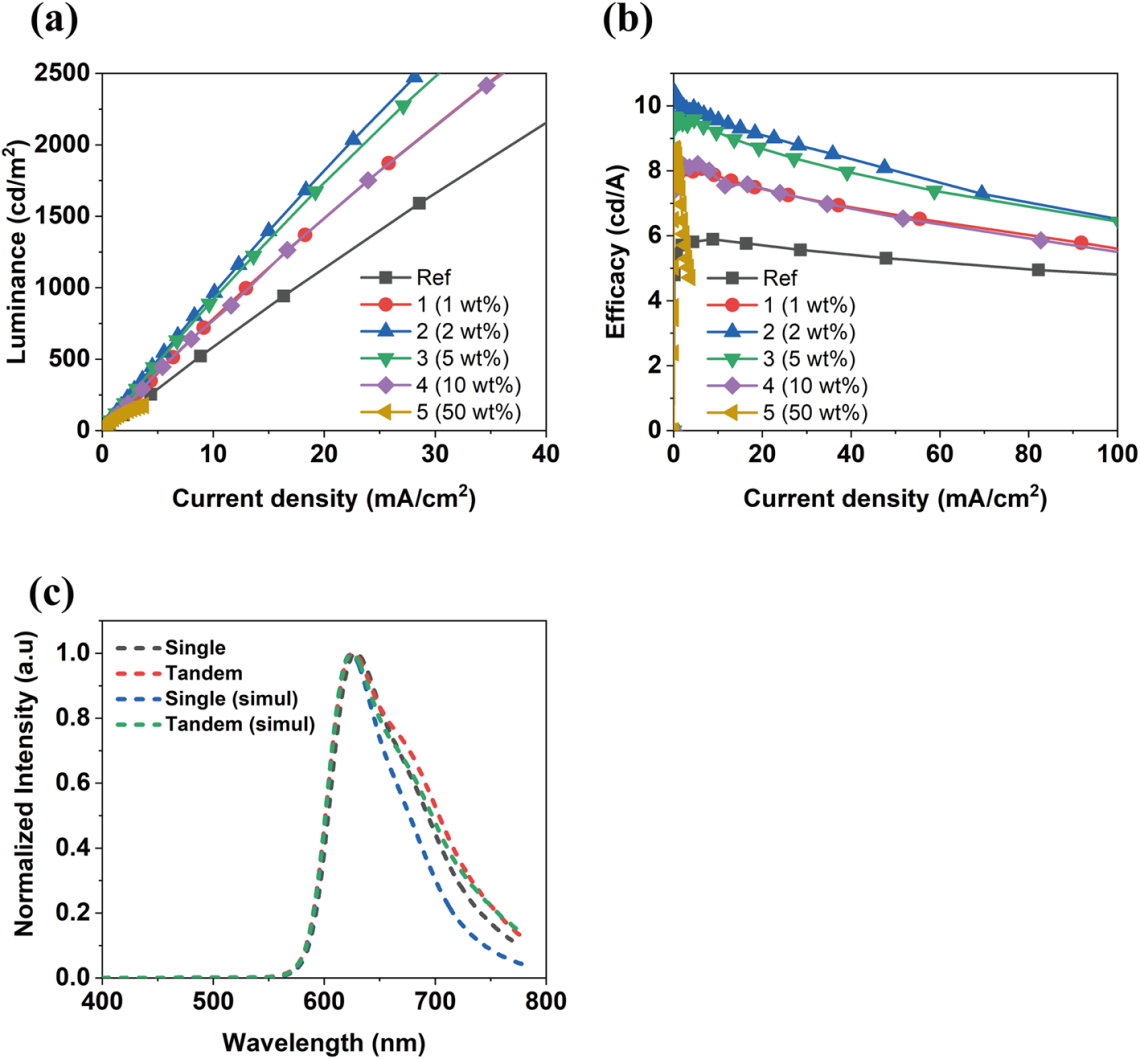
Device performance of non-cavity tandem OLEDs fabricated with various concentrations including a single OLED (reference): (**a**) Luminance-current density, (**b**) Efficacy-current density, and (**c**) Normalized EL spectra (experimental and simulation results).

On the other hand, LiH, an inorganic compound, is easily decomposed during vacuum deposition because of its simple chemical structure. However, device performance was greatly affected by the doping concentration. The performance of the devices was measured for each doping concentration to establish the ideal point shown in Fig. 1. As a result, LiH could function as an n-type dopant and easily transfer the charge to adjacent layers at a concentration of 2 wt% (see Fig. 1a,b).

**Figure 2:**
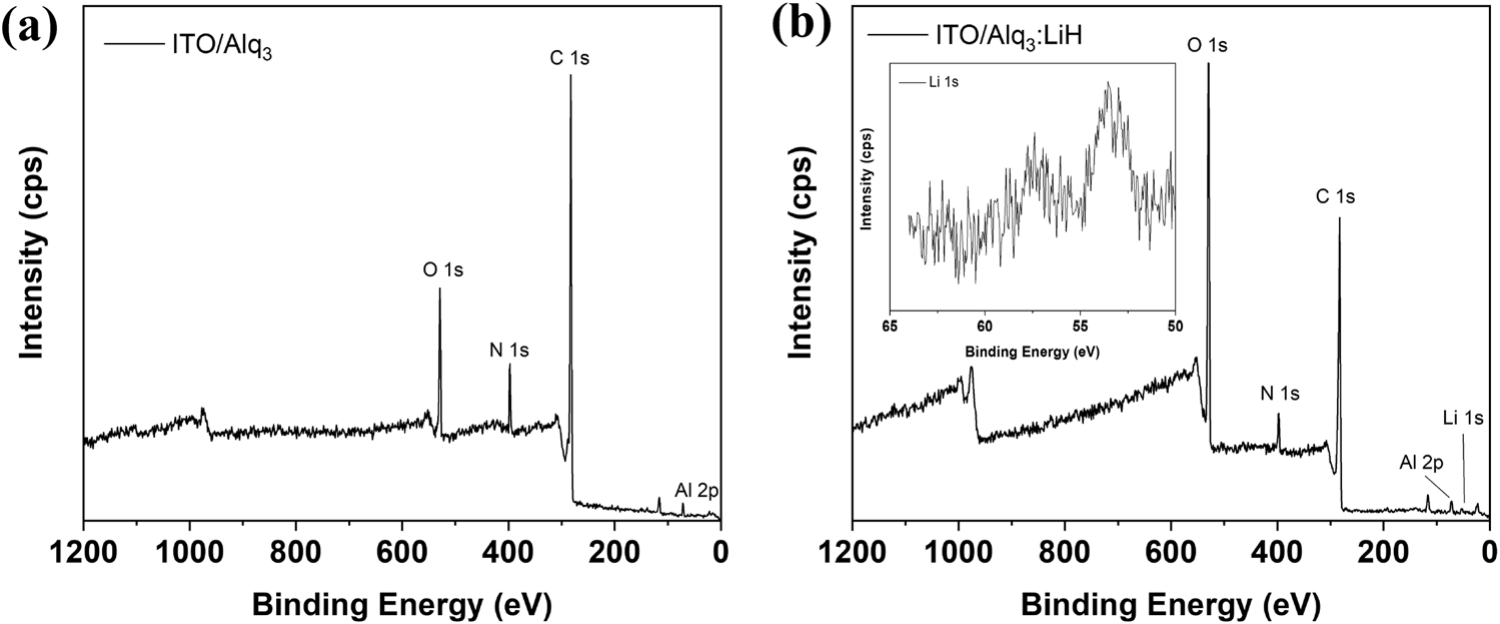
XPS spectra of vacuum-deposited thin film (40 nm): (**a**) Alq_3_ and (**b**) Alq_3_:LiH (2 wt%) on ITO-patterned glass substrate.

When deposited in a vacuum, it is widely known that LiH decomposes into Li and H_2_^37^. At this point, H_2_ is removed in a vacuum through purging of the chamber. Ultimately, the remaining Li will be deposited through evaporation. Figure 2 shows XPS spectra to confirm whether Li actually functions as an n-type dopant. When Alq_3_ and LiH were co-doped as a thin-film, a Li peak appeared in Fig. 2b compared to Fig. 2a. As shown in the inset in Fig. 2b, the Li 1 s core level was observed at approximately 54 eV. Hence, we can conclude that LiH acted as an n-type dopant when deposited with Alq_3_.

Figure 1c shows the experimental and simulation spectrum of the single and tandem devices, respectively. In general, in a device having a cavity structure, a spectral narrowing phenomenon occurs when a tandem structure is applied. Our tandem device has no spectrum narrowing due to its non-cavity structure, and shows different tendencies compared to that of a single device. The EL spectrum is represented by the product of the PL of the emitting layer (EML), Fabry–Perot (FP), and two-beam interference (TI) factor. The corresponding factors calculated by the simulation are depicted in Fig. S4. In the tandem device and the single device, opposite tendencies of FP and TI are formed in the long wavelength region. Accordingly, a cavity condition is formed on the long wavelength region side of the tandem device and a broad spectrum is observed, as shown in Fig. 1c.

### Fabrication of microcavity tandem NIR OLED

Based on the optimized CGL, a microcavity structure was used to fabricate NIR OLEDs. The theory of the microcavity effect is shown in Fig. S5. There are two main factors in the microcavity structure. Fabry–Perot equations and two-beam interference factors are expressed as follows:

1 $${f}_{FP}\left(\lambda \right)=\frac{{|{t}_{2}|}^{2}}{{(1-R)}^{2}+4R({\mathit{sin}\frac{\Delta {\phi }_{FP}}{2})}^{2}},$$

where $$\Delta {\phi }_{FP}=-{\phi }_{1}-{\phi }_{2}+n{k}_{0}2{d}_{2}$$ and $$R=|{r}_{1}|\cdot |{r}_{2}|\cdot {e}^{-\kappa {k}_{0}2{d}_{2}}$$,

2 $${f}_{TI}\left(\lambda ;{d}_{1}\right)=1{+(|{r}_{1}|\cdot {e}^{-\kappa {k}_{0}2{d}_{1}})}^{2}+2\cdot |{r}_{1}|\cdot {e}^{-\kappa {k}_{0}2{d}_{1}}\cdot \mathit{cos}\Delta {\phi }_{TI}$$

**Figure 3:**
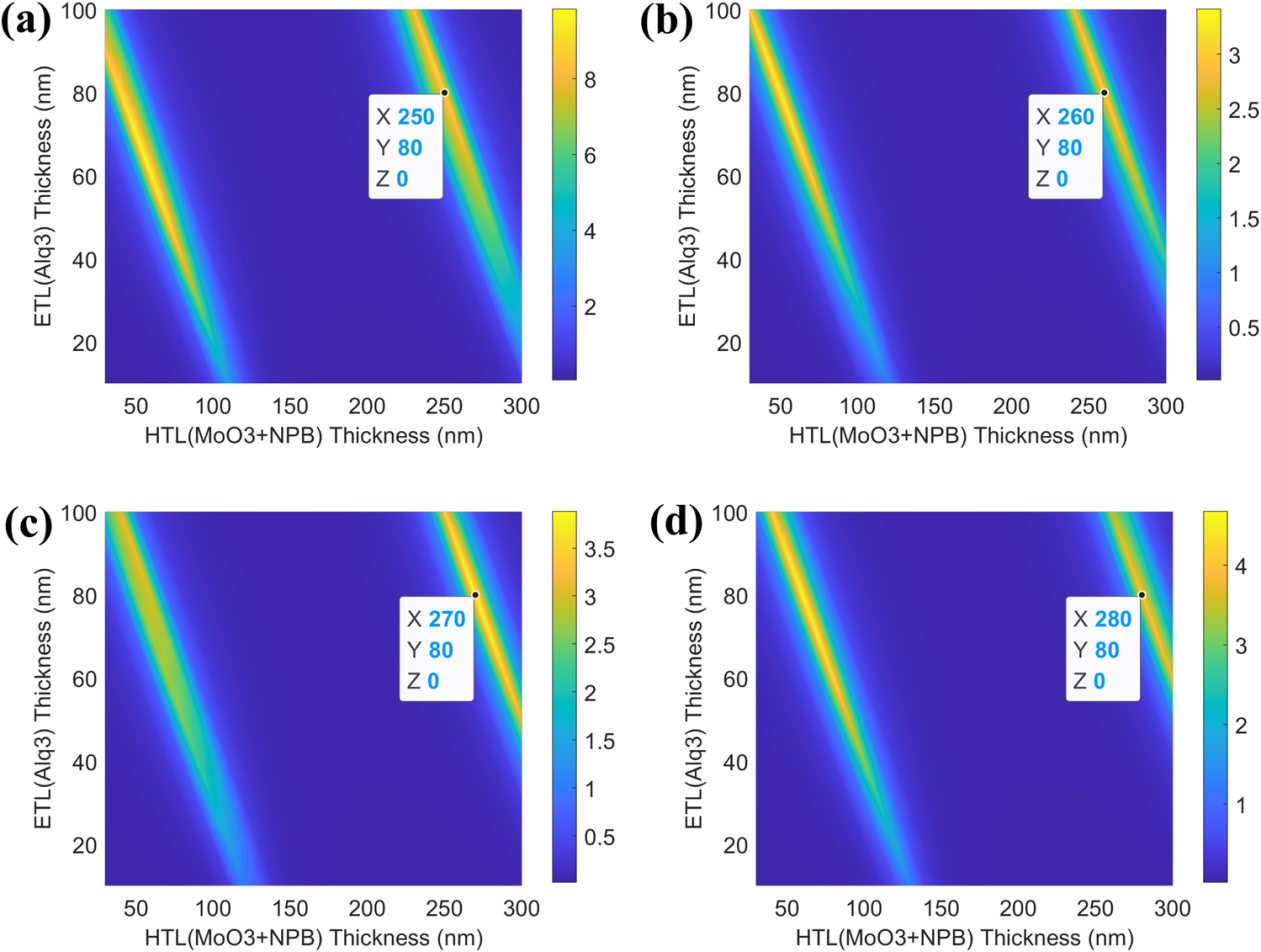
Optical simulation values of 1st and 2nd cavity order of maximum intensity depending on the thickness of HTL(MoO_3_ + NPB) and ETL(Alq_3_) at each target wavelength of NIR OLEDs: (**a**) 710 nm, (**b**) 730 nm, (**c**) 750 nm, and (**d**) 770 nm. All the figures were generated using MATLAB.

To fabricate a device with the desired wavelength, it is crucial to find the thickness of the organic layer where 2nd-order cavities occur^45–48^. Through optical simulation, the thicknesses of (MoO_3_ + NPB) serving as a hole transport layer (HTL) and Alq_3_ serving as an electron transport layer (ETL) were calculated where the 2nd-order cavities occurred, as shown in Fig. 3. There are two points where the 2nd order cavity occurs shown in Fig. S6. However, considering the charge balance and mobility, it is right to take the thickness of the hole injection longer. In a tandem structure, two layers of HTL and ETL exist, so each layer is constructed by stacking half the thickness obtained from the simulation. Therefore, half of the thickness of HTL (X) and ETL (Y), where 2nd-order cavity occurs at each target wavelength, were deposited on each layer, as shown in Fig. 3a–d. Table 1 presents the simulated values for each layer thickness of HTL and ETL for each target wavelength. Then, we calculated the EL intensity using each optimized layer thickness. Each EML has a Fabry–Perot factor and a two-beam interference factor (see Fig. S7). The cavity gain is obtained by independently adding the Fabry–Perot and two-beam interference factors present in each EML. (see Eqs. (3), (4), (5)) Finally, the EL spectra of the device is calculated by multiplying the cavity gain by the PL spectra of the emitter. (see Eq. (6))

**Table 1:** Thickness of organic material with maximum of 2nd-order cavity at each wavelength obtained through optical simulation. The thicknesses shown represent one layer each.

Target wavelength (nm)	HTL (MoO_3_ + NPB) (nm)	ETL (Alq_3_) (nm)
710	125	40
730	130	40
750	135	40
770	140	40

3 $${f}_{FP}\left(\lambda \right)={f}_{FP1}(\lambda )+{f}_{FP2}(\lambda )$$

4 $${f}_{TI}\left(\lambda \right)={f}_{TI1}(\lambda )+{f}_{TI2}(\lambda )$$

5 $${G}_{cav}\left(\lambda \right)={f}_{FP}(\lambda )\cdot {f}_{TI}(\lambda )$$

6 $$EL\_spectrum={G}_{cav}\left(\lambda \right)\times I\_spectrum$$

Simulation was also performed by changing the thickness of Ag as the anode at each wavelength to find the optimized structure. The simulation EL spectra of various conditions are shown in Fig. S8. As the Ag thickness was changed, the FWHM, maximum intensity, and radiance changed as shown in Table 2. Considering the FWHM, maximum intensity, and radiance, it was determined that the NIR OLED would have optimal performance with an Ag thickness of 20 nm or 30 nm. To compare the electrical and optical properties, microcavity single and tandem NIR OLEDs with the target wavelength of 730 nm were fabricated. The structure of single device A (named A(S)) was configured as follows: Ag (30 nm)/MoO_3_ (10 nm)/NPB (90 nm)/Bebq_2_ : Ir(piq)_3_ (30 nm, 8wt %)/Alq_3_ (40 nm)/Liq (1 nm)/Al (100 nm). Tandem devices B and C (named B(T) and C(T)) had the following configurations: Ag (x nm)/MoO_3_ (10 nm)/NPB (120 nm)/Bebq_2_ : Ir(piq)_3_ (30 nm, 8wt %)/Alq_3_ : LiH (40 nm, 2wt %)/MoO_3_ (10 nm)/NPB (120 nm)/Bebq_2_ : Ir(piq)_3_ (30 nm, 8wt %)/Alq_3_ : LiH (40 nm, 2wt %)/Liq (1 nm)/Al (100 nm); B(T) (x = 30 nm); C(T) (x = 20 nm). The energy level alignment and schematic structure of the microcavity tandem NIR OLED are depicted in Figs. S9 and S10.

**Table 2:** Simulation values according to the thickness of Ag.

Ag thickness (nm)	FWHM (nm)	Max. intensity (a.u)	Radiance (a.u)
15	60	13.85	119.2
20	30	18.06	99.40
30	19	18.38	59.34
40	14	11.93	29.85

### Electroluminescence performance of microcavity tandem NIR OLED

**Figure 4:**
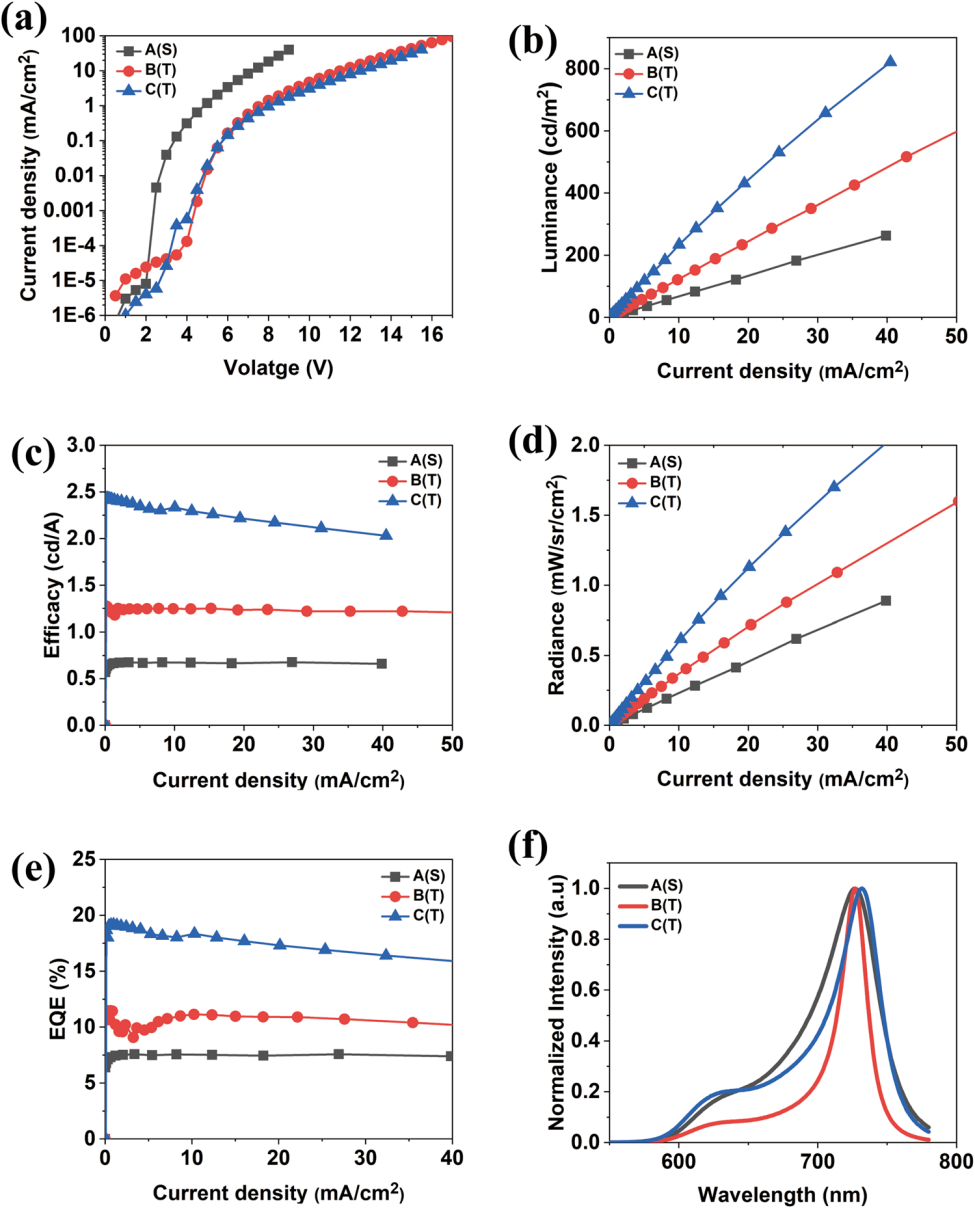
Device characteristics of the microcavity single (**a**) and tandem devices (**b**,**c**): (**a**) current density–voltage, (**b**) luminance-current density, (**c**) efficacy-current density, (**d**) radiance-current density, (**e**) EQE-current density, and (**f**) normalized EL spectra of the single and tandem NIR OLEDs.

The electrical properties of all devices at the same current density are shown in Fig. 4a–e; the numerical data under the same current density of 30 mA/cm^2^ are shown in Table 3. The normalized EL spectra with emission peaks of 727, 727, 732 nm for devices A to C are shown in Fig. 4f, respectively. Due to its tandem structure, the driving voltage of B(T) and C(T) is higher than that of A(S), but the electrical characteristics are more than 2 times as high. B(T) has the lowest narrow FWHM and excellent Q-factor, but it is importnat for the NIR OLED to be faricated to realize a stable device with high radiance for bio-medical applications. Hence, the radiance of the device was increased, and the stability was secured taking the Ag thickness to 20 nm (Fig. 4b–e). Finally, C(T) was fabricated and gave a high record of maximum EQE of 19.17% and a radiance emittance of 6.6 mW/cm^2^, which a achieves performance comparable to that of the reported NIR OLED^27,28,49^. To prove the stability and reliability of the best device (C(T)) and apply it in an in-vitro experiment, the lifetimes of the C(T) at 5 mW/cm^2^ were measured, and the results are shown in Fig. S11. The operational lifetime at 5 mW/cm^2^ for A(S) was extremely unstable because it drives at a high current density. On the other hand, C(T) had a half-lifetime of about 16 h because of its optimized structure. By utilizing a tandem structure, we eliminated the requirment for a large amount of current to generate a high radiant emittance in a single device.

**Table 3:** Comparison of electrical and optical values of NIR OLEDs at 30 mA/cm^2^.

Type of microcavity NIR OLEDs	FWHM (nm)	Luminance (cd/m^2)^	Efficacy (cd/A)	Radiance (mW/sr/cm^2)^	EQE (%)
Device A	52	182	0.676	0.618	7.58
Device B	23	350	1.22	1.09	10.7
Device C	34	657	2.11	1.7	16.9

### In-vitro experiment using microcavity tandem NIR OLED

Previously, a cell-based in-vitro experiment using a red OLED was reported by our group^20^. In addition to this, blue (B), green (G), red (R), and NIR OLEDs were used to irradiate human fibroblasts to identify the overall trends of cell cytotoxicity and proliferation in this study. To conduct a cell experiment, informed consent was obtained from all participants. The structure configurations of B, G, and R OLEDs are presented in the Method section. Normal human fibroblasts were sprayed onto a 96-well plate and irradiated with light at a distance of 1.5 cm using an OLED jig, as shown in Fig. S12. The OLED jig is configured to irradiate under a total of three conditions including control and designed to block light interference through a barrier between conditions. Cellular changes were observed in cells of the B, C, F, and G lines among all the cells irradiated on the 96-well plate, as shown in Fig. S13.

**Figure 5:**
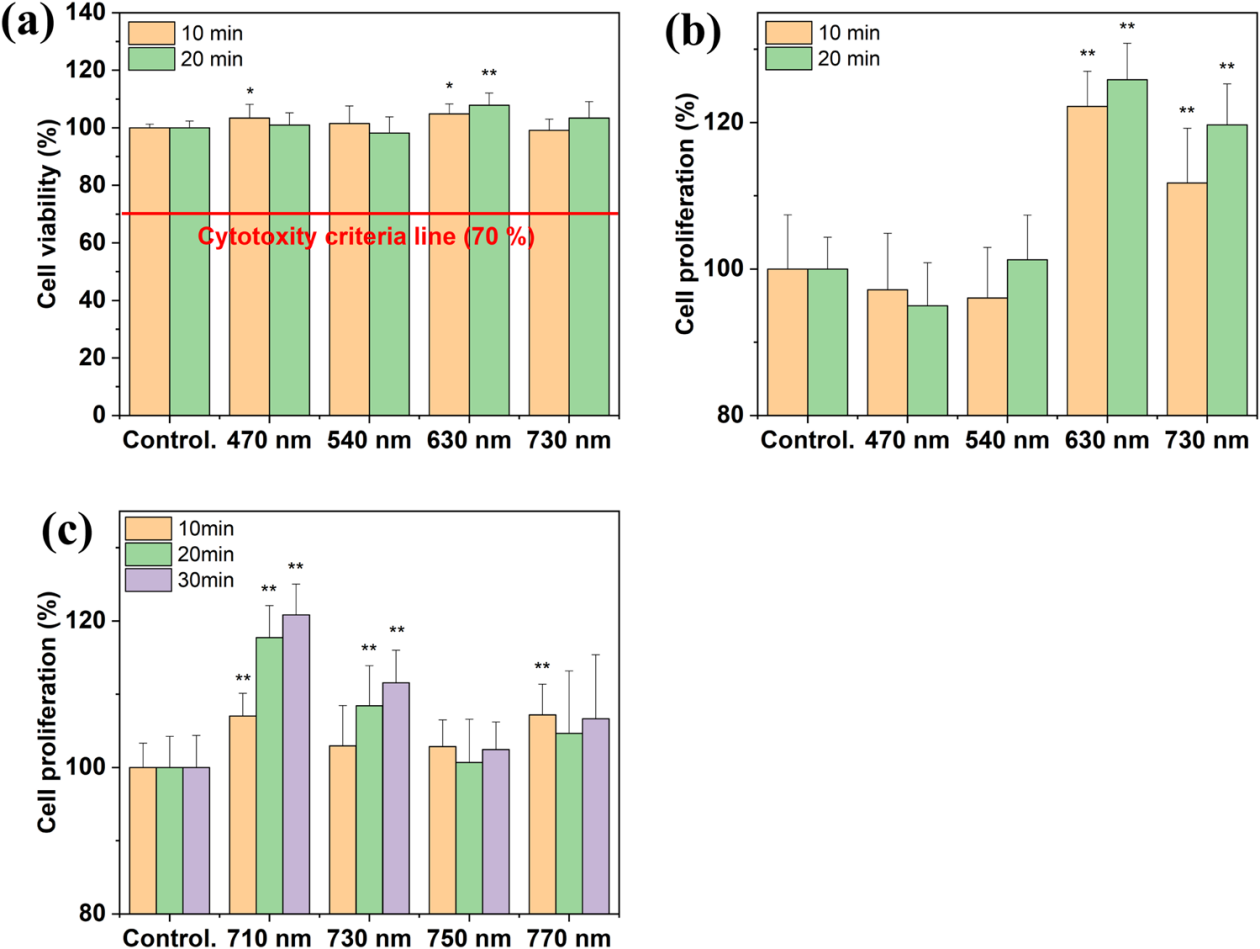
Cell effect in normal human fibroblasts (* $\le$ 0.05, ** $\le$ 0.01), values are mean percentage $\pm$ standard error of mean (n = 8): (**a**) cell viability and (**b**) cell proliferation over entire wavelength range. (**c**) cell proliferation in NIR wavelength range.

The first experiment was performed using B, G, R, and NIR OLEDs; the second observed the effects within the NIR region between 700 and 800 nm. First, B (470 nm), G (540 nm), R (630 nm), and NIR (730 nm) OLEDs were used with a radiant emittance of 5 mW/cm^2^ for 10 and 20 min to measure cell viability. The normalized EL spectrum of each device is shown in Fig. S14. According to ISO 10993-5 standards, if cell viability is more than 70% when cells are irradiated with light, it is judged that there is no cytotoxicity. Hence, there was no cell cytotoxicity at any wavelength, as shown in Fig. 5a. Then, cell proliferation was measured by the commonly used CCK-8 assay kit. Prior to evalutating the results of the proliferation, the heat dissipation of each device was monitored to validate whether the dissipated heat would affect the result. As a result, all devices were driven at a lower than the body temperature, and the influence of heat could be excluded shown in Fig. S15. The results showed that there was no cell proliferation effect in B or G. On the other hand, cell proliferation effects of about 26% were present when cells were irradiated with the red OLED for 20 min; that value was 20% when cells were irradiated with an NIR OLED for 20 min, as shown in Fig. 5b.

For the next experiment, the cell proliferation effect was observed in the same manner using NIR OLEDs with wavelengths of 710, 730, 750, and 770 nm fabricated in this study; each normalized EL spectrum is shown in Fig. S16. Considering the previously mentioned optical simulation and charge balance, we fabricated NIR OLEDs with each wavelength with variation of the HTL and ETL thickness. Using the same methods as in the first experiment, the cells were irradiated with a radiant emittance of 5 mW/cm^2^ for 10, 20, and 30 min. When cells were irradiated with the 710 nm NIR OLED, there was outstanding cell proliferation in comparison to the control; cell proliferation effects at the remaining wavelengths were insufficient. In particular, when the cells were irradiated for 30 min with 710 nm wavelength light, cell proliferation of about 21% was observed, as shown in Fig. 5c.

## Discussion

To apprehend the correlation between the wavelength and energy dose (radiant emittance $\times$ exposure time) of cell proliferation, we need to understand the wound healing mechanism. The mechanism of phototherapy has not yet been exactly clarified, and is still the subject of debate. The commonly known mechanism can be summarized as follows. When light stimulation is applied, adenosine triphosphate (ATP) is produced through the synthesis of deoxyribonucleic acid (DNA) and ribonucleic acid (RNA) in the mitochondria of the cells; NO is released, thereby promoting metabolic activity. Then, NADH, which serves as an electron donor for other materials, is oxidized and transports electrons, whereby H^+^ is actively transported from the mitochondria to the membrane due to the energy released. At this time, electrons are transferred between membranes by cytochrome c oxidase, a water-soluble protein, and the electrochemical gradient of H^+^ borders the mitochondrial inner membrane. The gradient diffuses from the membrane to the mitochondria and produces ATP^16,50^. The medium in each cell, which plays an important role in this process is widely known to be cytochrome c oxidase. Thus, it is vital to select an effective wavelength region considering the light absorption spectrum of cytochrome c oxidase of fibroblasts^51^. Moreover, optimization of the energy dose in light therapy is also an important determinant due to biphasic response. Adequate balance is crucial because insufficient radiant emittance or exposure time does not bring about a treatment effect, whereas excessive radiant emittance or exposure time has an inhibitory effect^16^. Therefore, proper wavelength and energy dose choices are important for maximizing effectiveness. There are several factors that affect light therapy mechnisms such as energy, time, radiant emittance, treatment interval, coherence, pulse, and wavelength. It is hard to conclude cleary what are the best conditions; thus, various conditions have to be considered.

**Figure 6:**
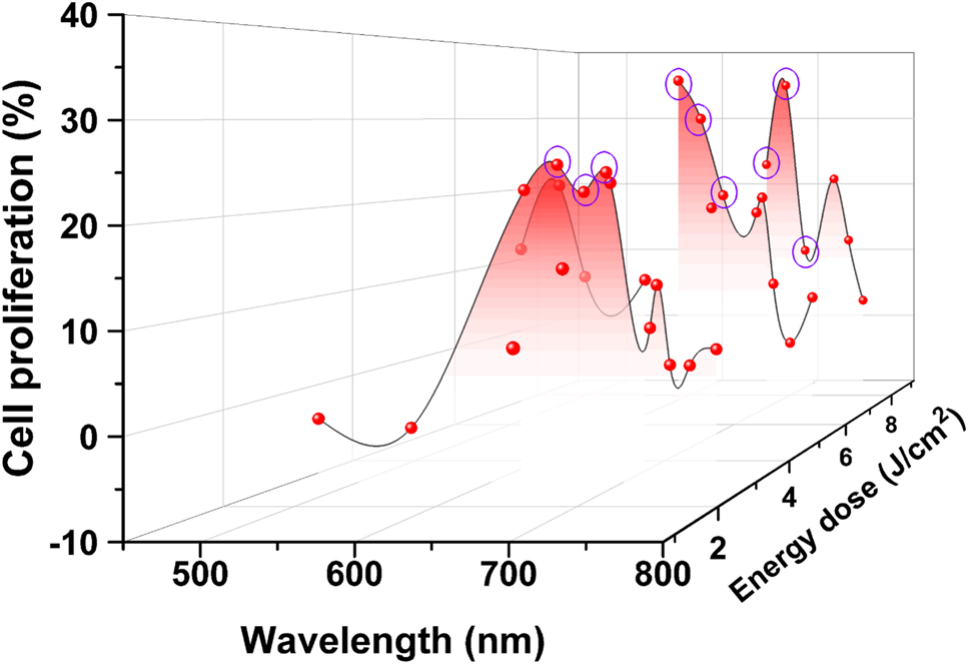
Tendency of cell proliferation for the entire wavelength region as a function of energy dose when irradiated by B, G, R, and NIR OLEDs. (Results indicated by purple circles are described in ref^20^.)

Our group previously performed a proliferation effect analysis within only the red region (630–690 nm)^20^. In addition, the effect of the wavelength and energy dose on cell proliferation was plotted shown in Fig. 6. Moreover, the tendency was confirmed at various wavelengths using NIR OLEDs fabricated in this study, including visible OLEDs (R, G, B).

By using visible and NIR OLEDs, it was shown that the red and NIR light affected the proliferation stage. In the B, and G regions, cell proliferation hardly appeared; however, a distinct cell proliferation effect was found in the red and early NIR regions. Moreover, it was confirmed that the tendency of cell proliferation clearly depended on the wavelength within the NIR region and that the proliferation effect rarely appeared at 750 nm. This tendency is somewhat similar to human fibroblast’s CCO light absorption spectrum^51^. The red and NIR regions showed trends similar to the absorption spectrum of cytochrome c oxidase; however, the B and G regions showed slightly different results. It is thought that the difference between B and G is caused by various factors, such as energy dose and penetration depth^16^.

## Conclusion

In summary, a highly reliable microcavity tandem NIR OLED that can be used in various practical biomedical applications has been fabricated. Human fibroblasts were irradiated with light with wavelengths ranging from visible to NIR to confirm the tendency of the cell proliferation effect. In particular, a cell proliferation effect was prominently found in the mid-late 600 nm (red) and early 700 nm (NIR) regions similar to CCO absorption spectrum. Although the cell proliferation effect in the NIR regions was not superior to that in the red region, the results are valuable because this light was actually found to penetrate deeply into the human skin and produce positive effects; it also has the advantage of invisibility. Moreover, it is meaningful in that this is the first study to report the trend within the entire wavelength using OLEDs.

## Methods

### OLED fabrication

The OLEDs were fabricated by vacuum deposition at 10^–6^ Torr using thermal evaporation equipment. Red, green, and blue OLEDs are commonly fabricated with Ag (30 nm) as an anode, MoO_3_ (5 nm) as a hole injection layer, Liq (1 nm) as an electron injection layer, and Al (100 nm) as a cathode at rates of 2, 0.5, 0.1, and 2 Å/s, respectively.

### Blue OLED

NPB as a hole transport layer (45 nm), 2-methyl-9,10-bis(naphthalen2-yl)anthracene (MADN) as a light-emitting layer (host), p-bis(p-N,N-diphenyl-aminostyryl) benzene (DSA-Ph) as a light-emitting layer (dopant, 25 nm, 3 wt%), and Alq_3_ as an electron transport layer (10 nm) were evaporated at a rate of 1, 3.01, 0.09, and 1 Å/s, respectively.

### Green OLED

NPB as a hole transport layer (50 nm), and Alq_3_ as a light-emitting layer and electron transport layer (50 nm) were evaporated at a rate of 1, and 1 Å/s, respectively.

### Red OLED

NPB as a hole transport layer (62 nm), Bebq_2_ as a light-emitting layer (host), and Ir(piq)_3_ as a light-emitting layer (dopant, 70 nm, 8 wt%) were evaporated at rates of 1, 3.31, and 0.25 Å/s, respectively. All of the OLEDs were encapsulated by a single layer of Al_2_O_3_ (30 nm) applied by atomic layer deposition. All organic materials and metals were purchased from Sigma-Aldrich and iTASCO, respectively.

### Device characterization

The electrical properties of the OLEDs were measured using a source meter (Keithley 2400, Keithley Inc.), and the optical properties were measured using a spectroradiometer (CS-2000, Konica Minolta Inc.). The operational lifetime of the OLEDs was measured under a constant current drive in air using a Si photodiode (Polaronix M9000S, McScience Inc.). The PL spectra of the red emitter were measured using a fluorescence spectrometer (FluoroMate FS-2, Sinco Inc.).

### Cell culture process

Human fibroblasts were extracted from human foreskins acquired during circumcision. Prior consent has been made from all subjects. All methods were carried out in compliance with with relevant guidelines and regulations. The study were approved by the Seoul National University Bundang Hospital Institutional Review Board (IRB Approval No. B-1603/340–309).

Skin specimens were handled using a procedure described in the literature^52^ but modified in the laboratory using thermolysin (P1512, Sigma, St. Louis, MO). Fibroblasts were grown in Dulbecco’s adapted Eagle’s medium (DMEM, LM001-05, WelGENE,Daegu, Republic of Korea) complemented with 10% fetal bovine serum (FBS, SH30919 (SH30071), HyClone by Thermo Scientific, Logan, UT), and 1 × antibiotic–antimycotic (15,240, Gibco by Life Technologies, Carlsbad, CA).

### Method of cell cytotoxicity measurement

A cell counting kit-8 (CCK-8, Dojindo, Kumamoto, Japan) assay was used to confirm the presence of OLED cytotoxicity. Standard human fibroblasts were seeded in 96-well plates at 3000 cells per well and cultured for 24 h. Then, cells were serum-deprived for another 24 h, after which they were irradiated with OLEDs at the specified dose. At that time, CCK-8 solution was applied to each well, and the samples were incubated for another 2 h. Absorption at wavelength of 450 nm was measured using a SPECTRAmax Plus384 microplate spectrophotometer (Molecular Devices, Sunnyvale, CA).

### Method of cell proliferation measurement

Standard human fibroblasts were seeded in 96-well plates at 2000 cells per well and cultured for 24 h. Then, cells were serum-deprived for another 24 h, followed by OLED irradiation at the suggested dose. At 72 h, CCK-8 solution was added to each well and incubated for another 2 h. Absorption at a wavelength of 450 nm was measured using a SPECTRAmax Plus384 microplate spectrophotometer.

### MATLAB simulation

The transfer matrix method model was used for the simulation, and the thickness of each layer except for the ETL and HTL was set. Exciton was generated in the middle within the EML layer, and the n and k values of all organic layers were measured through ellipsometry.

### Heat measurement

Heat dissipation was measured by FOTRIC 228GRD L28 (saferime). All devices were measure at 10 min intervals for 0–30 min under 5 mW/cm^2^ after setting a radiation rate of 0.8 and an ambient temperature of 20 °C.

## Supplementary Information

Supplementary Information.

## Data Availability

The datasets used and/or analyzed during the current study available from the corresponding author on reasonable request.
