# Supplementary material for: Cell proliferation effect of deep-penetrating microcavity tandem NIR OLEDs with therapeutic trend analysis
Source: Sci Rep. 2022 Jun 29;12:10935. doi: 10.1038/s41598-022-15197-4 (PMC9243069; doi:10.1038/s41598-022-15197-4)
Supplement: Supplementary file 1 — Supplementary Information. [file 41598_2022_15197_MOESM1_ESM.docx]

Supporting Information

**Cell Proliferation Effect of Deep-Penetrating Microcavity Tandem NIR OLEDs with Therapeutic Trend Analysis**

*Yongjin Park^1,4^, Hye-Ryung Choi^2,4^, Yongmin Jeon^3^, Hyuncheol Kim^1^, Jung Won Shin^2^, Chang-Hun Huh^2^, Kyoung-Chan Park^2^*, and Kyung-Cheol Choi^1^**

^1^ School of Electrical Engineering, Korea Advanced Institute of Science and Technology (KAIST), 291 Daehak-ro, Yuseong-gu, Daejeon 34141, Republic of Korea.

^2^ Department of Dermatology, Seoul National University College of Medicine, Seoul National University Bundang Hospital (SNUBH), Seongnam 13620, Republic of Korea.

^3^ Department of Biomedical Engineering, Gachon University, 1342 Seongnam-daero, Sujeong-gu, Seongnam-si 13120, Gyeonggi-do, Republic of Korea.

^4^ These authors contributed equally: *Yongjin Park and Hye-Ryung Choi*.

^*^ Corresponding author.

E-mail addresses: [gcpark@snu.ac.kr](mailto:gcpark@snu.ac.kr) (K. -C. Park), [kyungcc@kaist.ac.kr](mailto:kyungcc@kaist.ac.kr) (K. -C. Choi).

**Supplementary Figures**





**Figure S1.** Normalized PL spectra of a red emitter (Ir(piq)_3_) in a vacuum deposited thin-film (30 nm) at room temperature. The maximum peak wavelength of the dopant material is 633 nm ($\lambda$_ex_ = 400 nm).

**
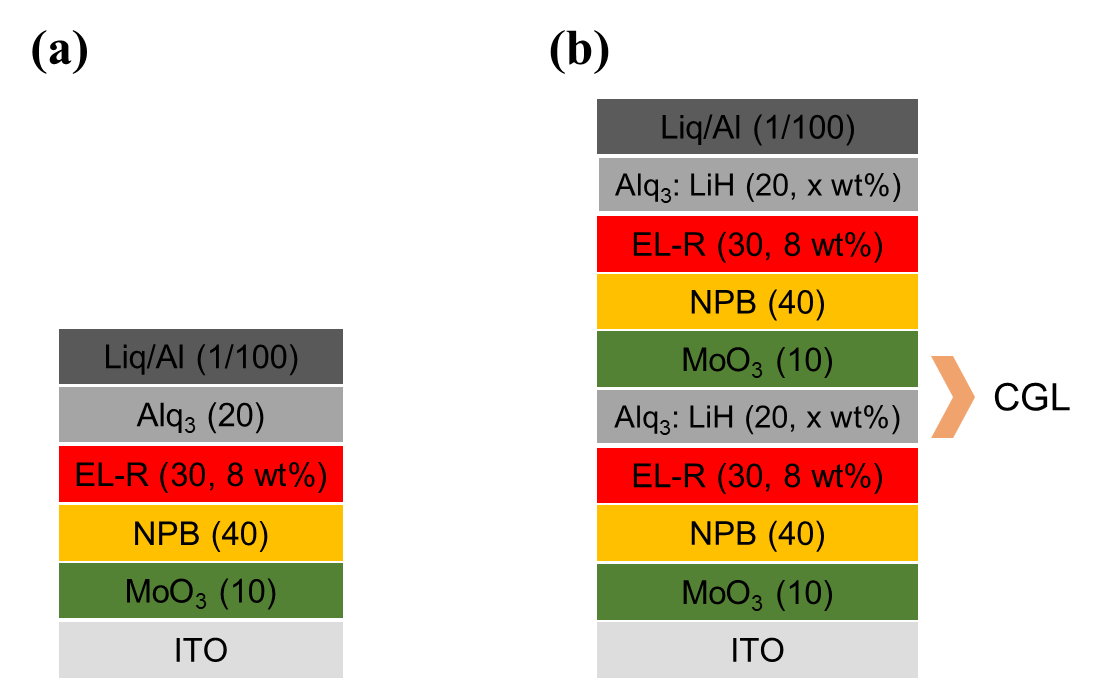
**

**Figure S2.** Schematic structures of non-cavity (a) Single, and (b) Tandem OLED.

**
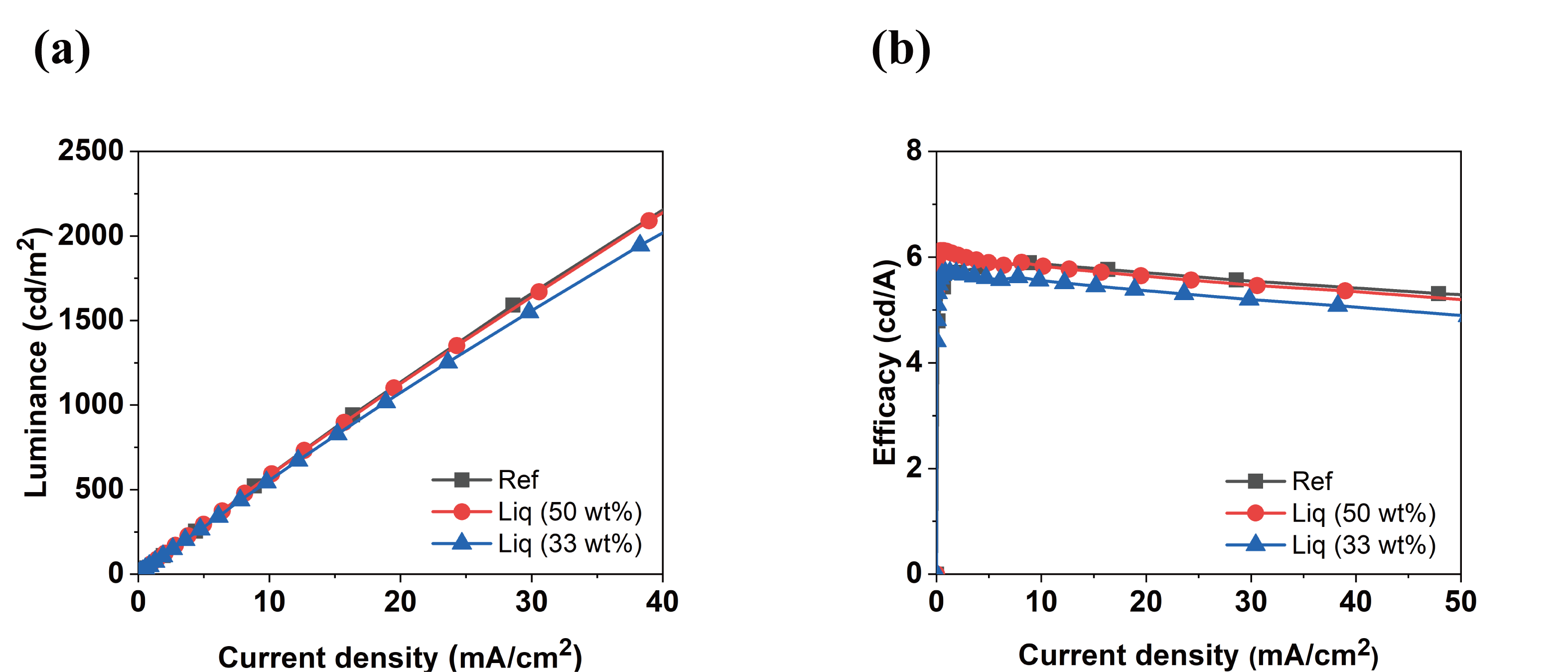
****Figure S3.** **Optical properties of non-cavity tandem OLEDs with an n-type dopant (Liq) and a single OLED (reference):** (a) Luminance vs current density, and (b) Efficacy vs current density.


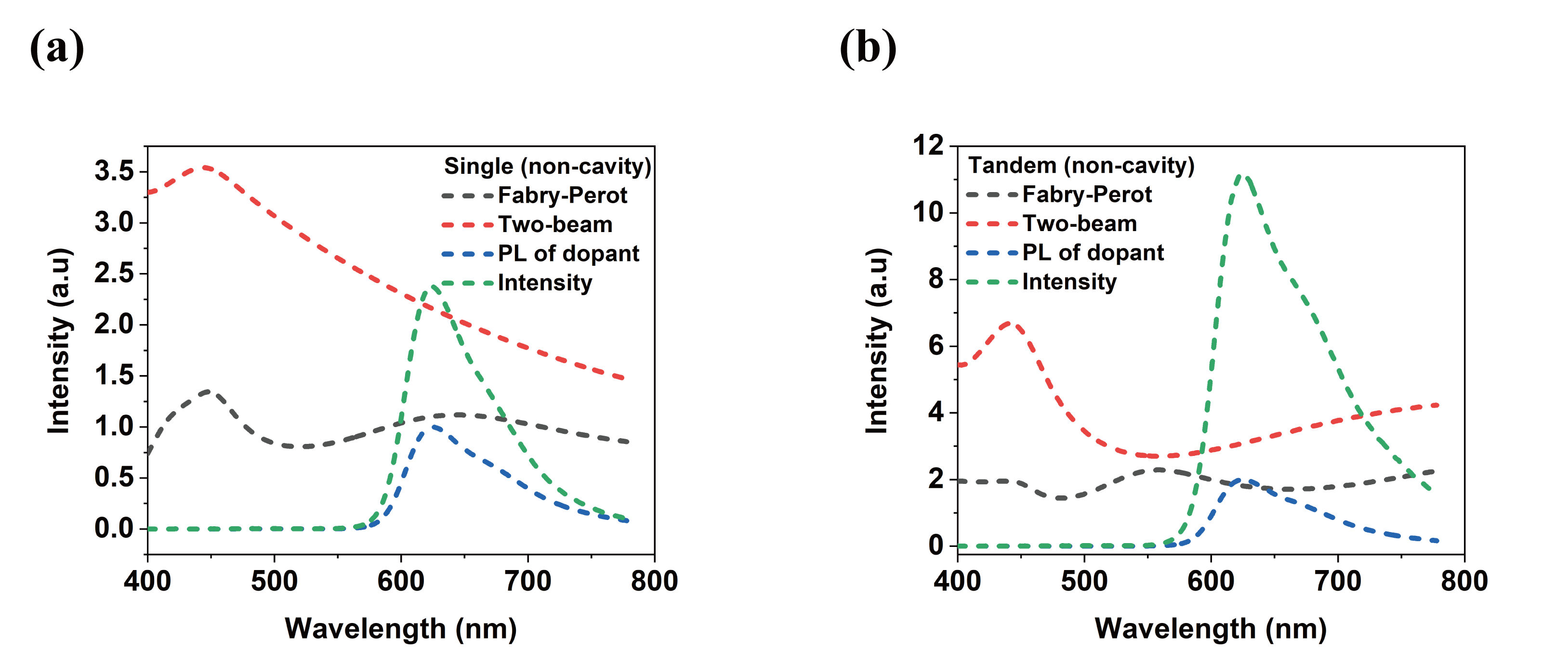


**Figure S4.** **Optical simulation of the EL intensity of non-cavity:** (a) Single, and (b) Tandem OLED. In the case of a tandem device, the two-beam factor is different, and the EL spectrum is slightly broad due to the weak-cavity effect occurring in the CGL.

**
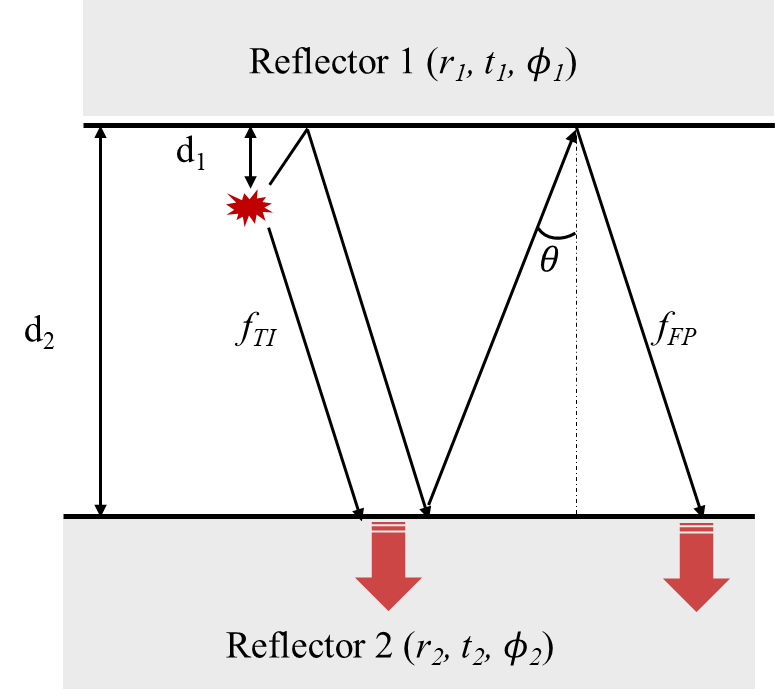
**

**Figure S5.** Light propagation in an OLED with microcavity effects (Fabry-Perot and two-beam interference). d_1_ is the distance between the formation position of exciton and cathode and d_2_ is the distance between the reflectors.


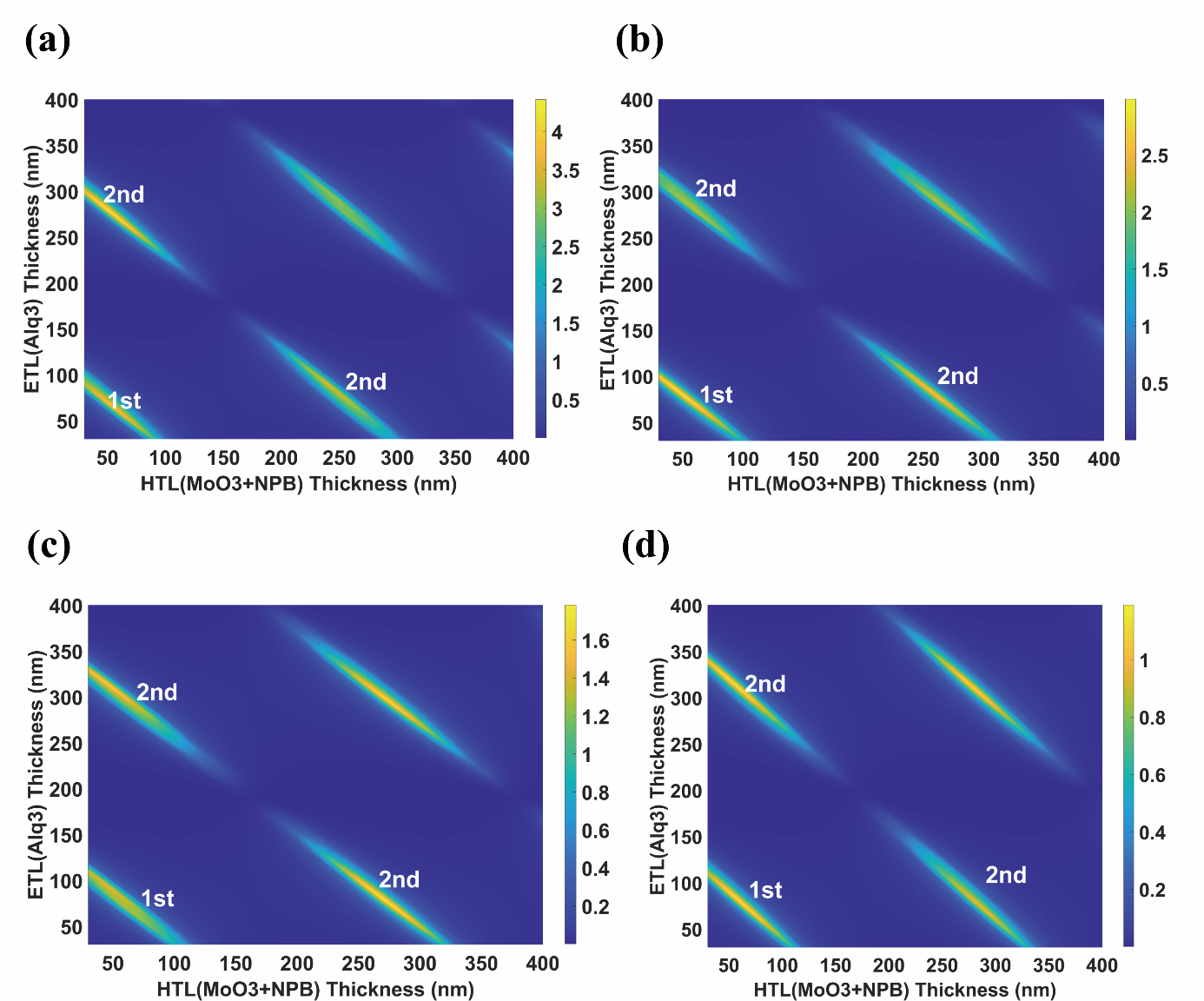


**Figure S6.** Emitted intensity of the cavity NIR OLED with a target wavelength of (a) 710 nm, (b) 730 nm, (c) 750 nm, and (d) 770 nm. There are two points of 2nd order cavity occurrence. The HTL and ETL thickness were varied from 30 to 400 nm. All figures were extracted from MATLAB.

**
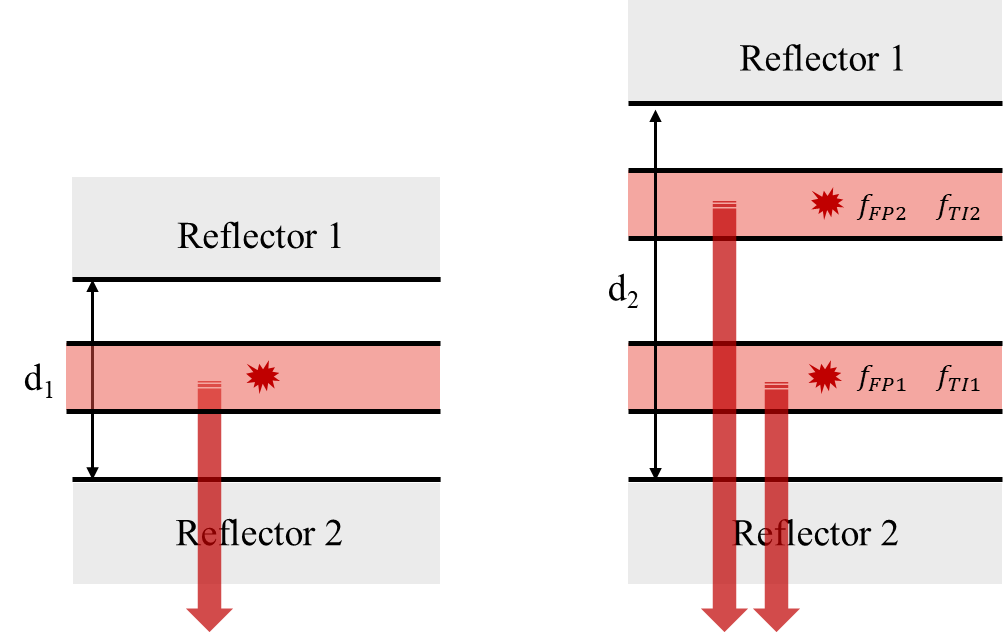
**

**Figure S7.** Optical simulation of a single and a tandem device using Fabry-Perot and a two-beam interference factor. d_1_ and d_2_ is the distance between both reflectors.





**Figure S8.** Optical simulation EL spectra of microcavity tandem NIR OLEDs depending on the thickness of anode (Ag). Considering intensity and FWHM, Ag thickness of 20 nm and 30 nm is the optimal point.


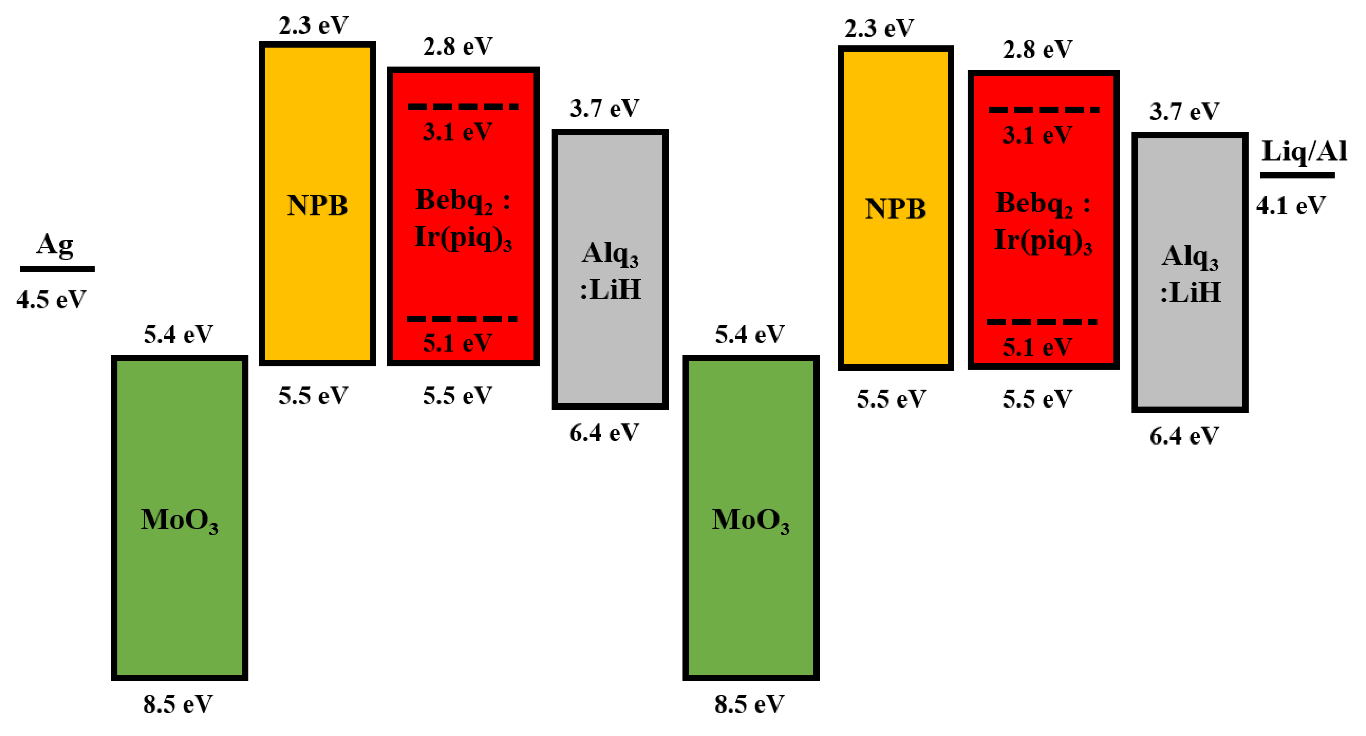


**Figure S9.** Structure configuration and energy level alignment of an optimized microcavity tandem NIR OLED.

**
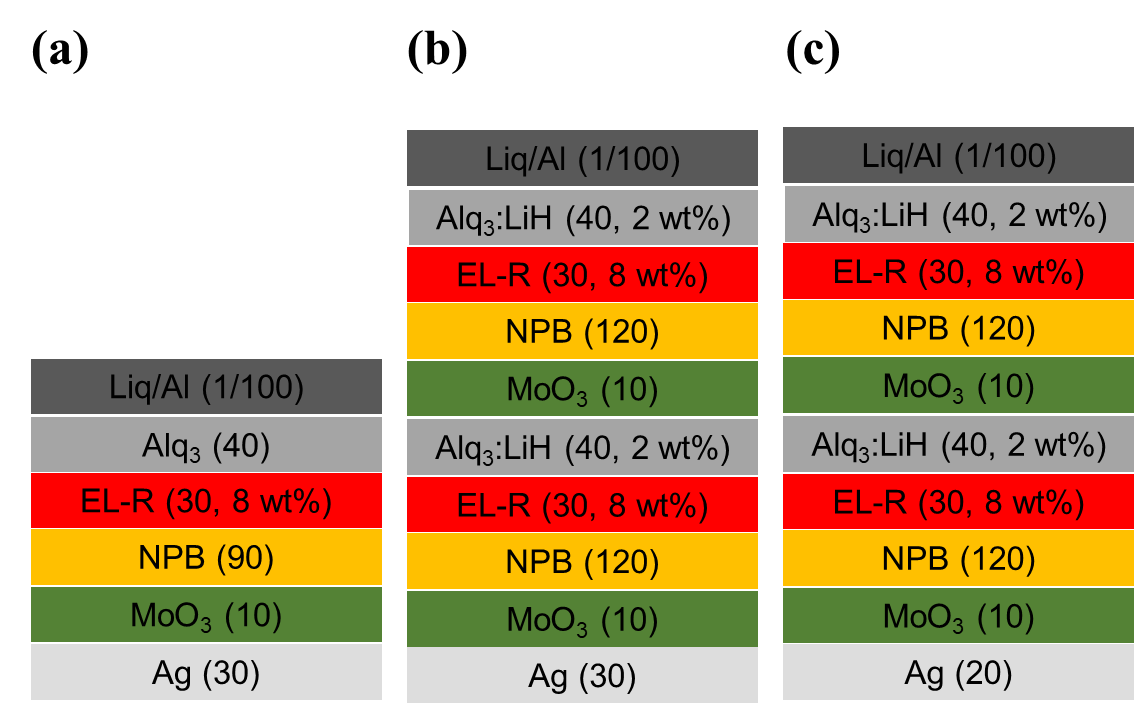
**

**Figure S10.** Schematic structures of the cavity (a) Single, (b) Tandem (Ag: 30 nm), and (c) Tandem (Ag: 20 nm) OLEDs.


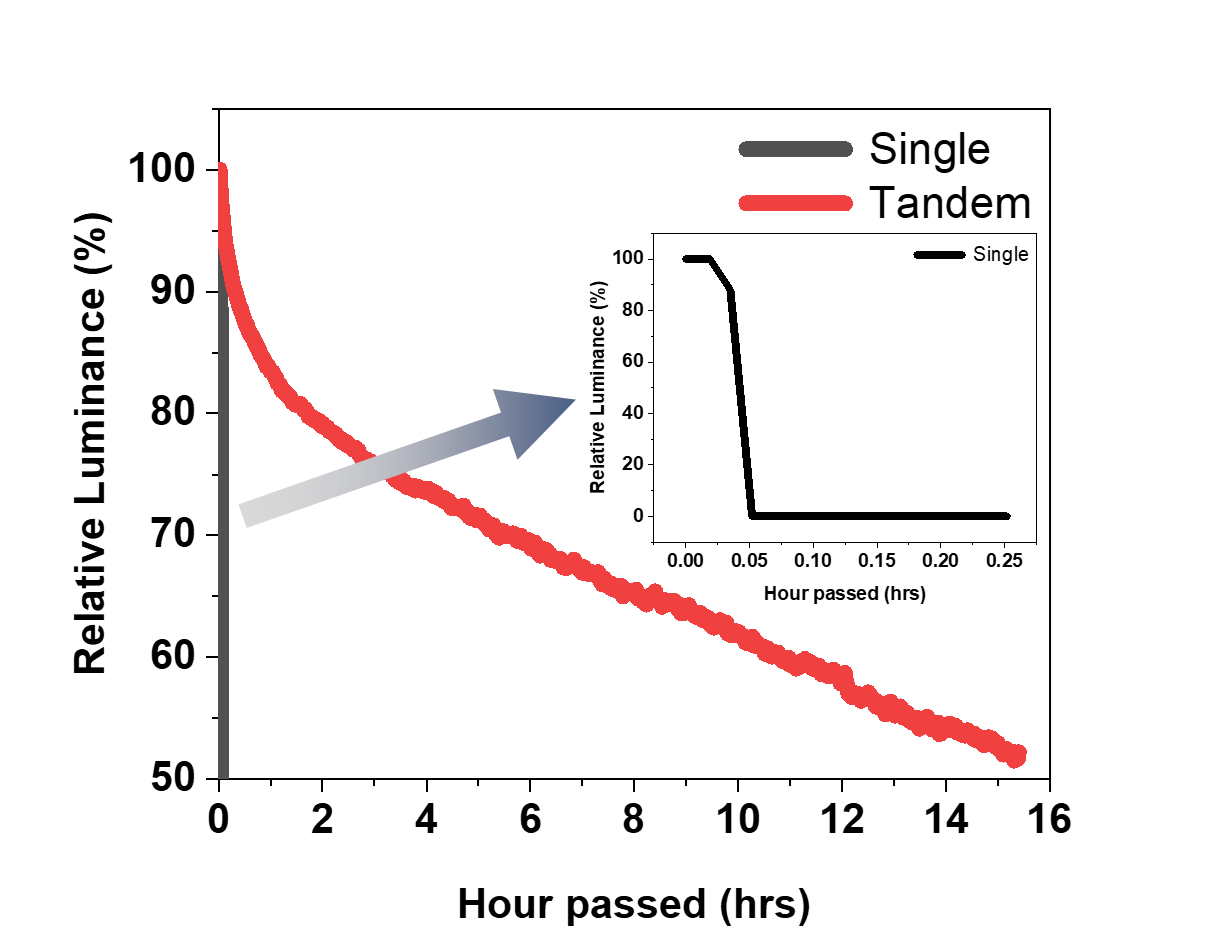


**Figure S11.** Operational lifetime of microcavity single and tandem NIR OLEDs at 5 mW/cm^2^.


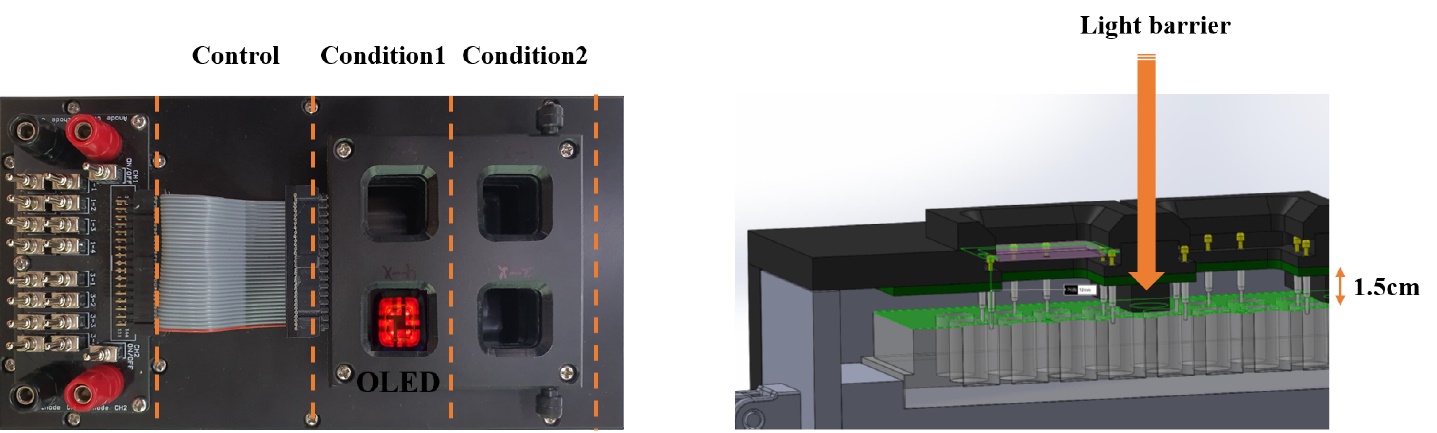


**Figure S12.** OLED jig designed to allow cell experimentation under three conditions (including control group). Figures were generated using Adobe illustrator 2022.


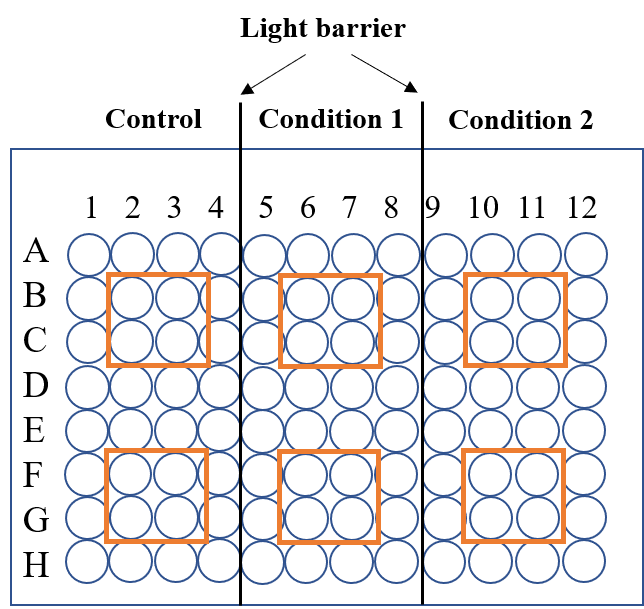


**Figure S13.** Schematic diagram of a 96-well plate used in the cell experiment. All cell lines were irradiated with light suitable for each condition, and the cell cytotoxicity and proliferation were confirmed by calculating the change of cells within each square.


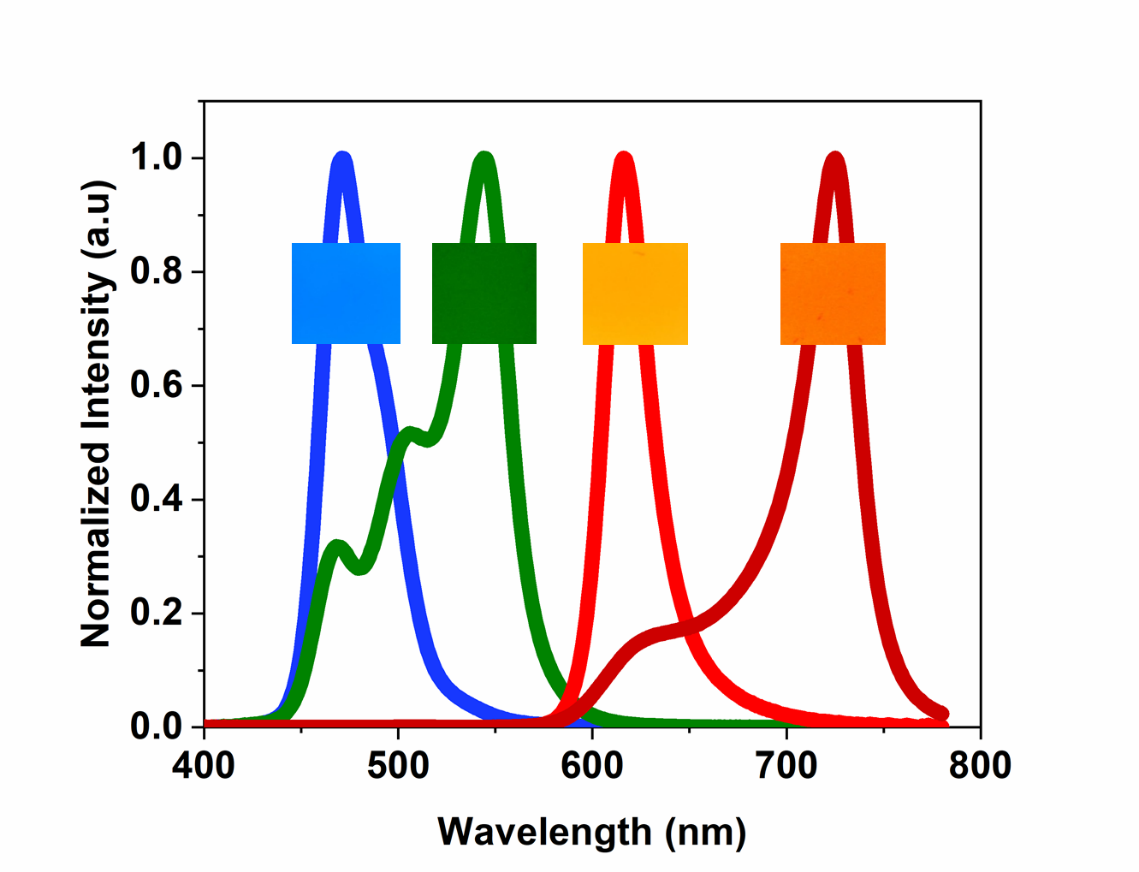


**Figure S14.** Normalized EL spectra of B, G, R, and NIR OLEDs.



**Figure S15.** Temperature changes over time measured by thermal imaging cameras. Heat dissipation of all OLEDs was measured at 10 min intervals at 5 mW/cm^2^.





**Figure S16.** Normalized EL spectra of NIR OLEDs with different wavelengths.
